# NML Computation Algorithms for Tree-Structured Multinomial Bayesian Networks

**DOI:** 10.1155/2007/90947

**Published:** 2008-01-20

**Authors:** Petri Kontkanen, Hannes Wettig, Petri Myllymäki

**Affiliations:** 1 Complex Systems Computation Group (CoSCo), Helsinki Institute for Information Technology (HIIT), P.O.Box 68 (Department of Computer Science), FIN-00014 University of Helsinki, Finland

## Abstract

Typical problems in bioinformatics involve large discrete datasets. Therefore, in order to apply statistical methods in such domains, it is important to develop efficient algorithms suitable for discrete data. The minimum description length (MDL) principle is a theoretically well-founded, general framework for performing statistical inference. The mathematical formalization of MDL is based on the normalized maximum likelihood (NML) distribution, which has several desirable theoretical properties. In the case of discrete data, straightforward computation of the NML distribution requires exponential time with respect to the sample size, since the definition involves a sum over all the possible data samples of a fixed size. In this paper, we first review some existing algorithms for efficient NML computation in the case of multinomial and naive Bayes model families. Then we proceed by extending these algorithms to more complex, tree-structured Bayesian networks.

## 

[1,2,3,4,5,6,7,8,9,10,11,12,13,14,15,16,17,18,19,20,21,22,23,24,25,26,27,28,29,30,31,32,33]
